# Allometric equations for estimating peak uprooting force of riparian vegetation

**DOI:** 10.3389/fpls.2023.1192486

**Published:** 2023-07-03

**Authors:** Yi Zhang, Wei Liu, Siming He

**Affiliations:** ^1^ Key Laboratory of Mountain Hazards and Surface Process, Institute of Mountain Hazards and Environment, Chinese Academy of Sciences, Chengdu, China; ^2^ School of Engineering Science, University of Chinese Academy of Sciences, Beijing, China

**Keywords:** riparian vegetation, uprooting force, uprooting test, flow resistance, allometric equations, morphological traits

## Abstract

Uprooting caused by flood events is a significant disturbance factor that affects the establishment, growth, and mortality of riparian vegetation. If the hydraulic drag force acting on riparian plants exceeds the peak uprooting force originate from their below-ground portion, it may result in the uprooting of these plants. Despite previous studies have documented and investigated the uprooting processes and factors influencing the peak uprooting force of plants, most of these studies have focused on how the root morphological traits of tree and shrub seedlings affect peak uprooting force or mainly collected data in indoor experiments, which may limit the extrapolation of the results to natural environments. To address these limitations, we assume that the peak uprooting force can be estimated by the morphological traits of the above-ground portion of the vegetation. In this study, we conducted *in-situ* vertical uprooting tests on three locally dominant species: *Conyza canadensis*, *Daucus carota*, and *Leonurus sibiricus*, in a typical riverine environment. The three species were found to have the highest abundance based on the outcomes of the quadrat method. We measured the peak uprooting force, plant height, stem basal diameter, shoot and root wet biomass, and shoot and root dry biomass of each plant and compared them between species. Furthermore, we quantified the influence of morphology on peak uprooting force. Our results showed significant differences in morphological traits and peak uprooting force among the three species. We found a significant positive correlation between peak uprooting force and the morphological traits of the three species. The peak uprooting force increases with plant size following a power law function which is analogous to allometric equations. The allometric equation provided a convenient and non-destructive method to estimate the peak uprooting force based on the above-ground morphological traits of the plants, which may help to overcome the limitations of measuring root morphological traits.

## 1 Introduction

Riparian zones serve as ecotones that bridge the transition between terrestrial and river ecosystems (Naiman and Decamps, 1997). These zones provide vital ecosystem services (Cole et al., 2020), including flood water retention, sediment transport, pollution control, nutrient sinks, and biodiversity conservation (Lowrance et al., 1985; Osborne and Kovacic, 1993; Tockner and Ward, 1999; Steiger et al., 2003; Burt et al., 2010; Liu et al., 2021; Tseng and Tinoco, 2021). In riparian zones, vegetation is an important component. It interacts with fluvial systems by affecting river hydrodynamics and morphodynamics (Perona et al., 2014). Riparian vegetation modifies flow field and turbulent structure and therefore influences sediment transport, sedimentation, and bedform formation (Nepf, 1999; Zong and Nepf, 2010; Yang and Nepf, 2019; Shan et al., 2020; Liu et al., 2021; Xu and Nepf, 2021). It also affects bank strength and modifies the soil condition such that it may increase bank stability (Pollen and Simon, 2005; Pollen-Bankhead and Simon, 2010; Krzeminska et al., 2019). In addition, large wood may play an essential role by protecting stream banks, reinforcing floodplains, and creating and stabilizing landscapes where new seeds begin to colonize (Gasser et al., 2019; Mao et al., 2020). All these factors affect the local morphodynamics and consequently the overall river morphology (Oorschot et al., 2016; Gonzalez Del Tanago et al., 2021). In turn, riparian vegetation is influenced by river-driven disturbances (e.g., flooding, droughts, groundwater fluctuations, etc.), which may cause the death of vegetation by scouring (Chen et al., 2012; Bywater-Reyes et al., 2015), uprooting (Bywater-Reyes et al., 2015; Bau and Perona, 2020; Piqué et al., 2020), burying (Kui and Stella, 2016), and desiccation (Barnes et al., 2013; Coughlan et al., 2018). Among these, uprooting is a major mechanism of riparian vegetation mortality that occurs when the hydraulic drag force exerted on the plant exceeds the resisting force provided by its roots (Bau and Perona, 2020).

Riparian plants exhibit two types of uprooting mechanisms during flooding: Type I is a flow-induced drag mechanism resulting in almost instantaneous uprooting when the drag force surpasses the peak uprooting force. Type II is a combination of flow and scour resulting in a decrease in the peak uprooting force, thus allowing the plant to be easily uprooted (Edmaier et al., 2011). For both uprooting mechanisms, it is important to determine the peak uprooting force of the vegetation, which is determined by a combination of root and soil characteristics. Several studies have explored the impact of root characteristics and soil conditions on peak uprooting force of vegetation. Bailey et al. (2002) found that root hairs had no significant difference in peak uprooting force by comparing samples of Arabidopsis thaliana with and without root hairs, and found that lateral roots and co-operation between roots play important roles on peak uprooting force. Root architecture seems to have an effect on peak uprooting force (Piqué et al., 2020). The heart- and tap-root system has a greater peak uprooting force than herringbone and plate root systems, regardless of the soil type (Dupuy et al., 2007). Schwarz et al. (2011) highlight the critical role of root diameter distribution for the prediction of the peak uprooting force of root bundles. They also highlight that soil water content and soil type affect root-soil friction, ultimately affecting the peak uprooting force of vegetation. Additionally, the sediment characteristics also influence the force necessary for uprooting. Finer sediment tends to impose greater uprooting forces compared to coarse sediment (Edmaier et al., 2014). This can be attributed to the fact that coarse sediments offer substantial resistance to root penetration due to their relatively larger particle size and higher substrate density (Handley and Davy, 2002; Dupuy et al., 2007).

Despite the importance of uprooting for riparian vegetation dynamics and fluvial geomorphology, few studies have quantified the peak uprooting force of riparian plants in natural settings. Some authors either focused only on peak uprooting force of the seedlings of trees and shrubs, or collected data in indoor experiments with uniform soil particles (Edmaier et al., 2011; Perona et al., 2012; Bywater-Reyes et al., 2015; Piqué et al., 2020), which may not reflect the variability and complexity of field conditions. In addition, some authors mainly focused on the relationship between root morphological traits and peak uprooting force, such as root biomass, root diameter, critical root length, and root length density (Bau et al., 2019; Piqué et al., 2020; Huai et al., 2021). These root morphological traits are challenging to measure, as traditional methods such as digging and minirhizotron tubes are time-consuming, labor-intensive, and uneconomical (Forzieri et al., 2010). They neglecting other traits such as above-ground biomass, stem basal diameter, and plant height that may also play a role and easy to measure. Henceforth, there is a pressing need for more extensive and authentic evaluations of the uprooting force of riparian vegetation in natural riverine habitats.

**Figure 1:**
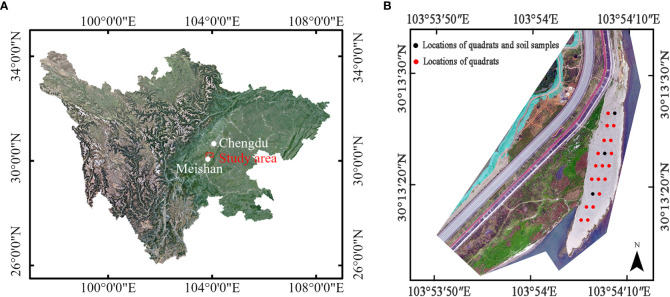
Study site of vertical uprooting tests: **(A)** location of study area relative to Chengdu city and Meishan City, Sichuan province; **(B)** Digital Orthophoto Map (DOM) of study area come from unmanned aerial vehicle (UAV) (2021.3), and the locations and arrangements of quadrats, marked as red and black solid circle, positions of soil samples, marked as black solid circle.

In this study, we aim to assess riparian herbaceous plants uprooting force in a river bar using a field vertical uprooting test. We also measured the morphological traits of the above-ground components of the vegetation. We assume that the peak uprooting force of the vegetation could be evaluated based on the morphological traits of its above-ground components. In our tests, three locally dominant species were selected as a research object. They occur near the river and are distributed throughout the whole riparian zone (Lite et al., 2005; Hagan et al., 2006). Herbaceous plants are more susceptible to flood disturbance, and exhibit a high species replacement rate at the lower elevations which are most susceptible to flooding (0–3 m) (Lyon and Sagers, 1998). Competitive annuals and flood-tolerant riparian herbs are also favored as pioneer species (Su et al., 2020). In July 2020, we observed a vegetation removal event on this bar during flooding, in which the flood removed all plants from the bar (**Figure 1B**). This is the reason why we chose this bar as the study site. In addition, uprooting test has proven to be one of the best methods to measure the uprooting force of the whole plant (Mickovski et al., 2005; Bywater-Reyes et al., 2015; Piqué et al., 2020).

In this work, 181 plant uprooting tests were performed in natural environments. We gauged plant morphological traits, including shoot height, stem diameter, shoot wet/dry biomass, and root wet/dry biomass, as well as the peak uprooting force of each plant. We compared the morphological traits and peak uprooting force between three herbaceous species and analyzed the effects of morphological traits on peak uprooting force. Allometric equations were established to estimate the peak uprooting force of plants based on aboveground morphological traits. The proposed allometric equation provides a fast and easy means of estimating peak uprooting force.

## 2 Materials and methods

### 2.1 Study site

The uprooting tests were carried out at a point bar (30°13′23.7″N, 103°54′8.47″E) in the Minjiang River on the southwest side of Chengdu, Sichuan province, China (**Figures 1A, B**). The bar is situated in a reach where we monitor the interaction between vegetation dynamics and floodplain morphology. The climate is humid subtropical monsoon, and the average annual temperature and rainfall are 16.8°C and 1,153.7 mm, respectively. The maximum rainfall occurs in July and August, with frequent rainstorms and other severe convective weather, which frequently causes river levels to rise. The bar is 200 m long, ranges from 15 m to 40 m wide, and its altitude varies from east to west, ranging from 418.2 m to 419.3 m. The south and east ends of the bar border the Minjiang River, while the west and north ends are adjacent to the floodplain. To investigate the soil particle distribution and water content at a shallow depth of 0–0.3 m belowground, we established three soil sample plots with the same volume of 0.09 m^3^. Note that a similar-sized volume was adopted by Meier et al. (2013) to collect fine sediment overlaying gravel. In **Figure 1B**, the three solid black circles represent the positions of soil samples #1 to #3, from north to south. The results of the soil particle distribution are displayed in **Supplementary Figure 1**. The soil primarily comprises gravel and cobbles, with a d_50_ value of 40 mm. Particles with diameters between 5 mm and 100 mm make up at least 92% of the soil sample. For soil materials smaller than 2 mm in diameter, the proportion increases with distance from the Minjiang River. The water content of soil samples #1 to #3 is 55.1%, 53.6%, and 48.9%, respectively, indicating a slight decrease in water content with distance from the river.

### 2.2 The investigation of plant diversity on the bar

In May 2021, we employed the quadrat method to investigate the plant diversity on the bar. This method has been used in prior studies, such as those conducted by Su et al. (2020) and Zeng et al. (2019), to assess the diversity of riparian herbaceous vegetation. We established a sample size of 2 m × 2 m, in line with Su et al. (2020). A total of 19 plots were demarcated on the bar. In each plot, we tallied and identified all plant species, and recorded their morphological traits such as plant height and stem diameter. Additionally, we documented the midpoint coordinates of each sample square, and marked them by black and red solid circles in **Figure 1B**. The results indicate that the bar was dominated by *Conyza canadensis* (CC), *Daucus carota* (DC), and *Leonurus sibiricus* (LS), which collectively accounted for 86.7% of the samples, with relative frequencies of 50%, 19.7%, and 17%, respectively. The remaining species only account for 13.4% of the samples. Due to their high abundance, only the dominant species, CC, DC, and LS, were selected as research objects for the vertical uprooting tests.

During the vertical uprooting test, the three species were observed at varying growth stages. CC was at a juvenile stage, DC was flowering, and LS was mature but not flowering (as shown in **Supplementary Figure 2**). CC had a fibrous or fleshy and short taproot, with many well-branched first-order lateral roots growing alongside it. The aerial part of DC had a central stem and an abundance of long lateral branches. Its taproot was long and fibrous, with only a few short first-order lateral roots. LS and CC each had a stem with uniformly distributed leaves along its length. LS had a branched and fibrous primary root, along with a mass of well-branched first-order lateral roots (as depicted in **Supplementary Figure 2C**).

### 2.3 *In-situ* uprooting tests

*In-situ* vertical uprooting tests were conducted from May 10 to 25, 2021. Samples of CC, DC, and LS with a stem length greater than 15 cm were selected as only samples of this length or greater can be fastened to a threaded rod using steel wire (**Supplementary Figure 3**). In accordance with Cabal et al. (2020), a minimum distance of 20 cm between two plants was maintained to avoid any vegetation interaction. A total of 54, 67, and 60 samples were collected for CC, DC, and LS, respectively.

Prior to commencing the vertical uprooting test, we obtained measurements of the stem diameters and plant heights using calipers with an accuracy of 0.02 mm and a tapeline with an accuracy of 1 mm. Following this, plant stems were cut 15 cm above the ground, and a threaded rod was affixed to the residual stems *via* a thread ring nut (**Supplementary Figure 3**). The thread ring nut was then connected to an uprooting mechanism (**Supplementary Figure 3**), which is analogous to those utilized in comparable studies (Bankhead et al., 2017; Piqué et al., 2020). The entire plant was subsequently uprooted at a consistent vertical velocity (5 cm/s, the speed of the electric winch).

After uprooting each plant, the shoot wet biomass was measured using a digital scale (8,200 g * 100 mg, Entris^®^ II Essential Line Precision Balance). To prevent internal moisture evaporation, the plant was sealed in a plastic bag, numbered, and the next plant was uprooted. At the end of the day, the plants were transported to the laboratory for further analysis. The roots were watered with running water and air-dried for 2 hours to allow for surface moisture evaporation. Subsequently, the root wet biomass was measured using a digital scale. Finally, the shoot and root parts of each plant were placed separately in an oven to dry at 70°C until a constant biomass was achieved. Then, the shoot and root dry biomass was quantified using a digital scale (8200 g * 100 mg or 210 g * 1 mg, Entris^®^, dry biomass less than 1 g was quantified using the latter). We extracted two variates from the force-time graph, including the total time required to pull out a plant ( $T_{total}$ , s) and the peak uprooting force ( $F_{R}$ , N).

### 2.4 Statistical analysis

We conducted a Shapiro-Wilk test to check for normal distribution of all morphological traits and pullout characteristics for the three species. To assess the relationship between morphological traits and pullout characteristics, we conducted Spearman correlation analysis. Additionally, we estimated the binary relationship between other morphological traits and $T_{total}$ and peak uprooting force while control for one morphological trait using zero-order correlation and partial correlation (Spearman correlation). We calculated the 95% confidence interval using the estimated mean of correlation coefficients and the standard error (SE). The goodness of fit of the relationship between peak uprooting force and morphological traits was evaluated using the coefficient of determination (R^2^) and the coefficient of significance at a 95% confidence level. All analyses were performed using R-studio statistical software (RStudio Team, 2015) version 2021.09.2.382, R version 4.1.2 (2021-11-01) (Team, 2013), the “ggplot2” R package (Wickham, 2016), and the “ppcor” R package (Kim, 2015).

### 2.5 Critical uprooting flow velocities

Riparian vegetation can increase water flow resistance during floods. The drag force of submerged vegetation can be estimated by:


(1) $$F_{D}=0.5\rho C_{D}A_{f}U^{2}$$

where ρ is the density of water, $C_{D}$ is the drag coefficient, $A_{f}$ is projected vertical frontal area of vegetation submerged in water, and U is the approach velocity (Hoerner, 1965). Once the drag force exceeds the peak uprooting force, the plant may be uprooted from the soil (Edmaier et al., 2011; Piqué et al., 2020). The primary factor governing the maximum force required to uproot a plant is predominantly linked to the characteristics of its root system. Prior research (Bailey et al., 2002; Schwarz et al., 2011; Gibson, 2012; Bau et al., 2019) has revealed that attributes such as the number of roots, root length, root shape, and root physiology significantly impact the uprooting force. However, gathering data on root system traits is a daunting task, given the roots’ invisibility. Thus, the ability to predict the peak uprooting force based on the aboveground vegetation’s morphological traits is crucial. Because the aboveground morphological traits are easily measurable compared to root traits. Allometric function is commonly used to express the relationship between plant characters. It can be linear or nonlinear, and can have different coefficients depending on environmental factors or phylogenetic groups (Jost, 2017). We presume that the allometric relationship between the height of vegetation and the maximum force required to uproot it follows a power law function:


(2) $$F_{R}=\;a_{1}\cdot H^{b_{1}},$$

where H is plant height, $F_{R}$ is the peak uprooting force of the vegetation, and $a_{1}$ and $b_{1}$ are coefficients of power law fitting. We presume a similar allometric equation between total frontal area and plant height:


(3) $$A_{f}=\;a_{2}\cdot {\text{H}}^{b_{2}},$$

where $a_{2}$ and $b_{2}$ are coefficients of power law fitting. Replacing $A_{f}\;$ in Equation (1) with Equation (3), and equating $F_{R}=\;F_{D}$ , we can calculate the approach velocity U when the submerged vegetation was uprooted:


(4) $$0.5\rho C_{D}\cdot a_{2}\cdot {\text{H}}^{b_{2}}\cdot U^{2}=\;a_{1}\cdot H^{b_{1}},$$

and defining the approach velocity U as critical flow velocity $U_{c}$ , we obtain:


(5) $$0.5\rho C_{D}\cdot a_{2}\cdot {\text{H}}^{b_{2}}\cdot {U_{c}}^{2}=\;a_{1}\cdot H^{b_{1}},$$

and


(6) $$U_{c}=\;\sqrt{2ac\cdot H^{b},}$$

and in which the coefficient a = $\frac{a_{1}}{a_{2}}$ , b = $b_{1\;}-b_{2}$ , $c=\;\frac{1}{\rho C_{D}}$ . For plants in a certain environment, the coefficients a, b, and c have a constant value. Therefore, we can easily and quickly estimate the $U_{c}$ based on vegetation height. It is also possible to replace plant height with any other morphological traits of the aboveground portions of the vegetation. It is noteworthy that equation (6) pertains solely to submerged vegetation type I uprooting.

## 3 Results

### 3.1 Uprooting force for individual plants

**Figure 2:**
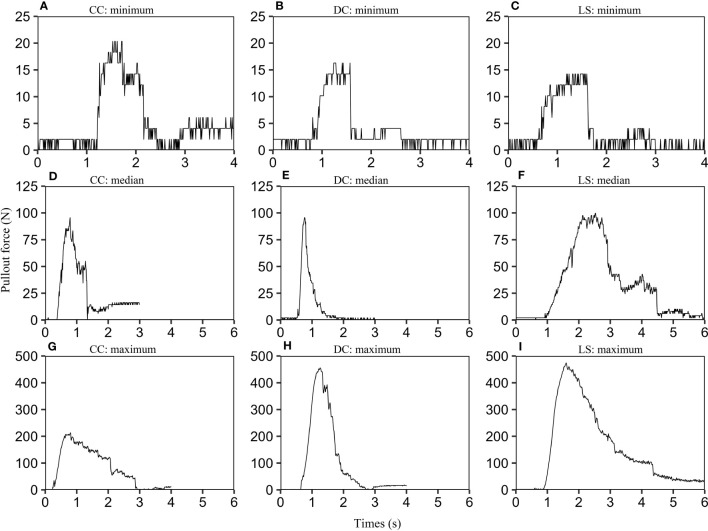
Uprooting force-time graphs for CC, DC, and LS. For each species, we selected three plant samples according to the following protocol: firstly, we calculated the maximum pullout force for all CC samples using their force-time graphs. We then arranged the maximum pullout forces in ascending order and identified the minimum, median, and maximum values. Finally, we selected the corresponding samples for these three values and labeled them as CC: minimum **(A)**, CC: median **(D)**, and CC: maximum **(G)**. The same selection criteria were applied to DC **(B, E, H)** and LS species **(C, F, I)**. In order to limit the range of the time axis, any invalid data located at the beginning or end of each curve was removed.

For each species, we calculated the maximum of pullout force according to force-time graphs of all samples, and rank the maximum of pullout forces in ascending order, and find the minimum, the median, and the maximum. The samples corresponding to these three values are selected. The pullout force-time graphs for these samples are depicted in **Figure 2**. The nine graphs exhibit a similar form consisting of three stages: firstly, a linear elastic stage where the uprooting force increases proportionally with time, and is primarily governed by the elastic properties of the soil and roots. Secondly, a non-linear stage occurs due to the gradual activation of the root-soil interface friction, ultimately resulting in the peak uprooting force (Schwarz et al., 2010). Finally, in the third stage, the uprooting force diminishes over time due to the gradual breakage and slippage of roots from the soil (Bailey et al., 2002). The three phases are consistent with what has been observed in previous studies (Edmaier et al., 2014; Bau and Perona, 2020). We also observed multiple force drops subsequent to the peak uprooting force. These force drops are likely due to the fracturing of taproots and abundant lateral roots at varying intervals (Bailey et al., 2002; Edmaier et al., 2014; Piqué et al., 2020).

### 3.2 Uprooting characteristics and morphological traits

Initially, we assessed the normality of six morphological traits and two pullout characteristics using the Shapiro-Wilk test for each species. Our findings indicated that only plant height of LS, stem diameter of CC and LS, peak pullout force of CC, and total time of DC were sourced from a population with a Gaussian distribution (p > 0.05). The remaining variables did not pass the normality test (p< 0.05). In the following, we compare each variable between species according to its median value rather than its mean value. **Table 1** presents the range, median, and standard deviation of six morphological traits and two pullout characteristics, $F_{R}$ and $T_{total}$ . The morphological traits include stem diameter, plant height, shoot wet and dry biomass, and root wet and dry biomass. Our observations indicate that the standard deviations of root wet and dry biomass and shoot wet and dry biomass in three species exceed the median value. Additionally, other traits and the two pullout characteristics also exhibit standard deviations greater than a quarter of the median. These findings suggest that the eight parameters exhibit considerable intra-specific variations. One potential source of morphological variation is natural variation. Plants demonstrate inherent diversity in their form and structure, which can arise from genetic, environmental, or developmental factors. For instance, mutations, gene expression, and environmental stimuli (such as light, temperature, and water availability) may yield distinct phenotypes among individuals (Kalisz and Kramer, 2008; Borrelli et al., 2009).

**Table 1:** The range, median, and standard deviation (SD) of six morphological traits and two pullout characteristics for all samples of CC, DC, and LS.

		CC			DC			LS	
Parameters	Range	Median	SD	Range	Median	SD	Range	Median	SD
Plant height (cm)	23-78	41.5	13.7	53-152	88	24.1	23-150	84.5	29.1
Stem height (cm)	3.6-13.7	7.8	2.7	3.5-12.5	6.7	2.2	1.8-13.4	7.5	2.5
Root wet biomass (g)	1.2-48.2	7.1	**9.7**	0.8-67.5	7.7	**11.5**	1.3-116.2	13.1	**18.5**
Shoot wet biomass (g)	4.9-153	32.6	**34.9**	14.4-820	82.2	**186.9**	1.3-336.5	45.3	**70.8**
Root dry biomass (g)	0.3-11.3	1.9	**2.2**	0.4-22	2.3	**3.9**	0.3-21.1	3	**3.5**
Shoot dry biomass (g)	1-49.3	6.2	**10.5**	3.3-222.3	16.9	**49.7**	0.5-92.7	10.9	**21.3**
Peak pullout force (N)	22.4-213.6	97.7	46.6	16.3-455.7	97.7	94.1	16.3-476.1	98.7	**111.7**
Total times (s)	0.5-4.2	2	0.9	0.4-4.1	1.7	0.7	1-8.7	2.9	1.3

SD means standard deviation.

The standard deviation above the median has been bolded.

Compared to DC and LS, CC had the smallest plant height, root wet and dry biomass, and shoot wet and dry biomass, but the largest stem diameter, and a similar $F_{R}$ (median = 97.7 N, 97.7 N, 98.7 N for CC, DC, and LS). The highest median of $T_{total}$ was observed in LS (median = 2.9 s), while the lowest was observed in DC (median = 1.7 s). In this study, the plants were uprooted from the soil at a constant vertical velocity; therefore, the magnitude of $T_{total}$ is proportion to the root displacement. Root displacement can be affected by the number of lateral roots and root length (Hamza et al., 2007); hence, this may account for the interspecific variation of $T_{total}$ .

### 3.3 Correlations between morphological traits

**Figure 3:**
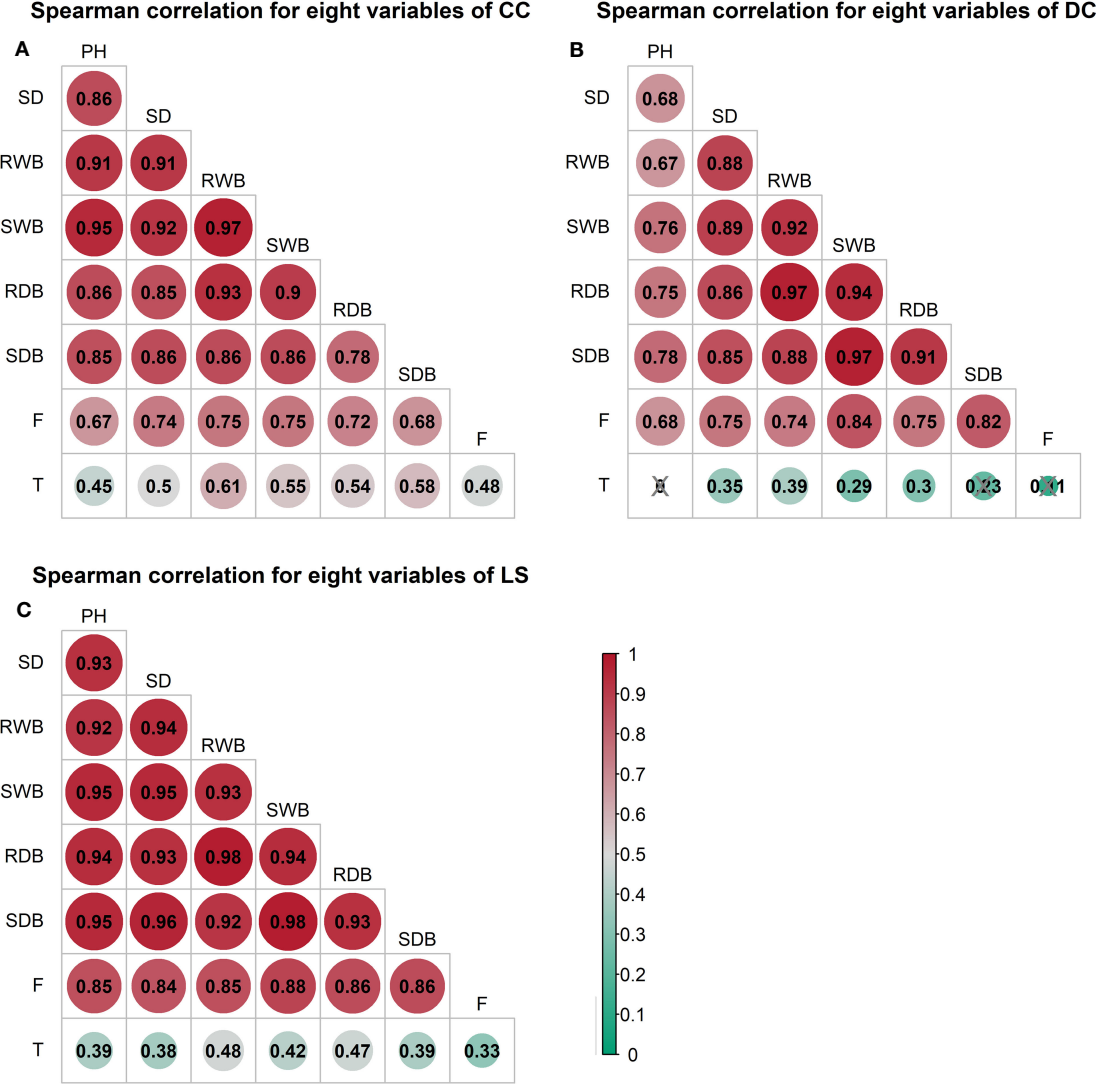
Spearman correlation analysis between morphological traits and pullout characteristics of three species: **(A)** CC **(B)** DC **(C)** LS. The number of samples for CC, DC, and LS is 54, 67, and 60, respectively. The size of the circle represents the magnitude of correlation coefficient $r_{s}$ . The circle filled with color from red ( $r_{s}=1$ ) to green ( $r_{s}=0$ ). Correlation coefficient $r_{s}$ is marked in each circle and the symbol ‘×’ represents non-significant *p*< 0.05. T = Total time, F = peak uprooting force, SDB/SWB = shoot dry/wet biomass, RDB/RWB = root dry/wet biomass, SD = stem diameter, PH = plant height.

**Figure 4:**
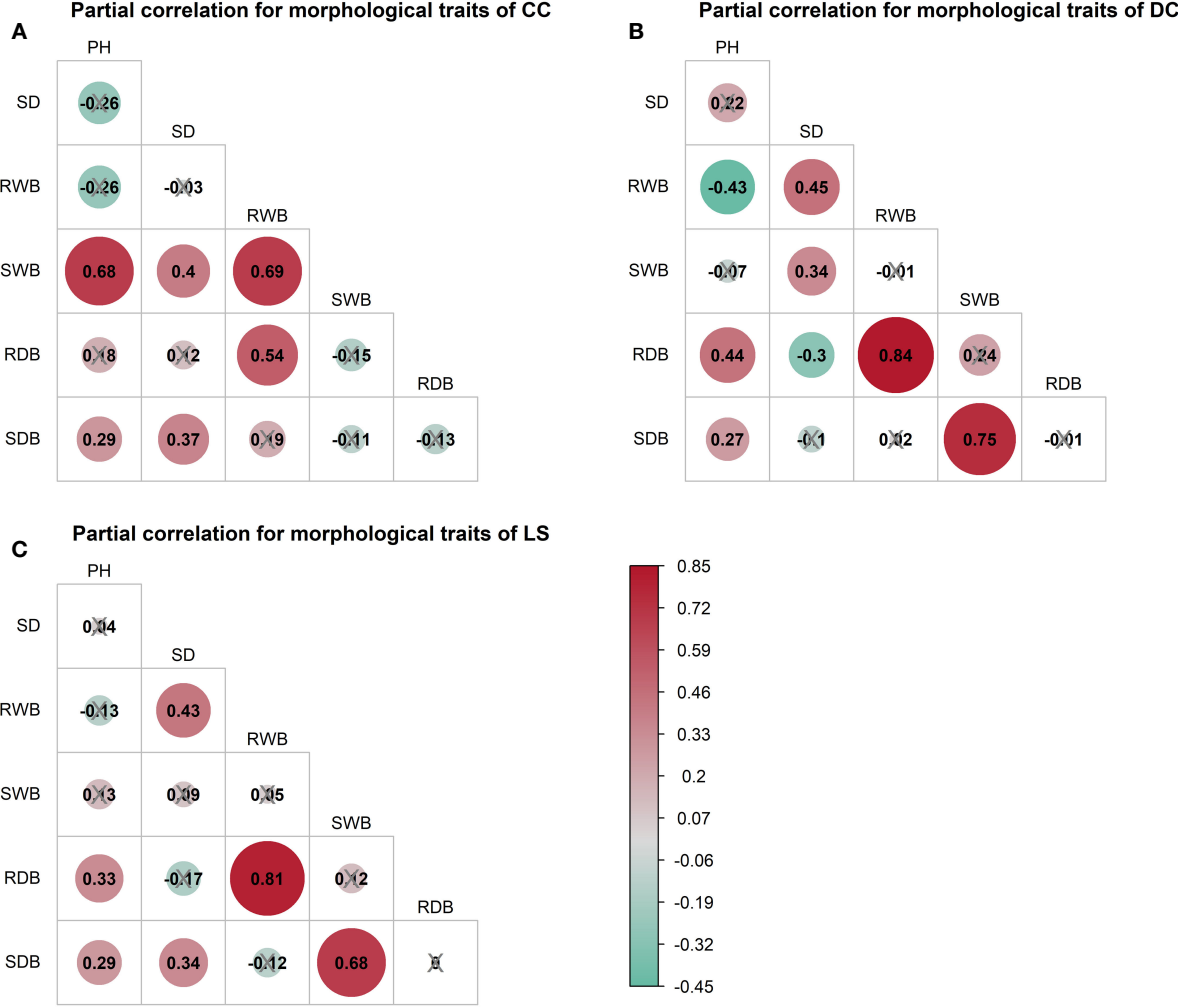
Partial spearman correlations among morphological traits of three species, **(A)** CC, **(B)** DC, **(C)** LS. The correlation of paired morphological traits is calculated by controlling effects of other morphological traits. Symbol ‘×’ represents non-significant *p*< 0.05. Correlation coefficient $r_{s}$ is marked in each plot and filled with color from red ( $r_{s}=0.85$ ) to green ( $r_{s}=-0.45$ ). SDB/SWB = shoot dry/wet biomass, RDB/RWB = root dry/wet biomass, SD = stem diameter, PH = plant height. The intensity of colors and numbers indicate the strength of the correlation.

The outcomes of the Spearman correlation analysis among morphological traits are illustrated in **Figure 3**. The dimensions of the circles signify the magnitude of the correlation coefficient $r_{s}$ , and the circles are filled with color from red ( $r_{s}=1$ ) to green ( $r_{s}=0$ ). The correlation coefficient $r_{s}$ is labeled in each circle, and the symbol “×” signifies a non-significant value of p > 0.05. Notably, a statistically significant positive correlation ( $r_{s}>0.67$ ) was observed between any two variables of the morphological traits (**Figure 3**). Furthermore, a correlation coefficient $r_{s}$ > 0.9 was observed between certain morphological traits of CC and DC (**Figures 3A, B**), as well as between the shoot wet biomass and root wet biomass. Similarly, a correlation coefficient $r_{s}$ > 0.9 was detected in all morphological traits for LS (**Figure 3C**). To investigate the distinct relationship between two morphological traits while controlling the potential influence of other traits, we conducted partial correlation analysis, the results of which are presented in **Figure 4**. The relationships between root wet biomass and root dry biomass, and between shoot dry biomass and shoot wet biomass, exhibited significant positive correlation in DC and LS (**Figures 4B, C**) even after controlling for the effects of other morphological traits, indicating that they were not affected by the other traits. On the other hand, the relationships between plant height and shoot wet biomass, between root wet biomass with shoot wet biomass, and root dry biomass, still displayed significant positive correlation for CC, which diverges from the findings of DC and LS. In addition, we observed that the correlations between most paired morphological traits lost their significance when we accounted for the effects of other morphological traits, implying that they are highly influenced by other morphological traits. This might be a probable explanation for their notable variability across different morphological traits and plant species.

### 3.4 Correlations between morphological traits and pullout characteristics

**Figure 5:**
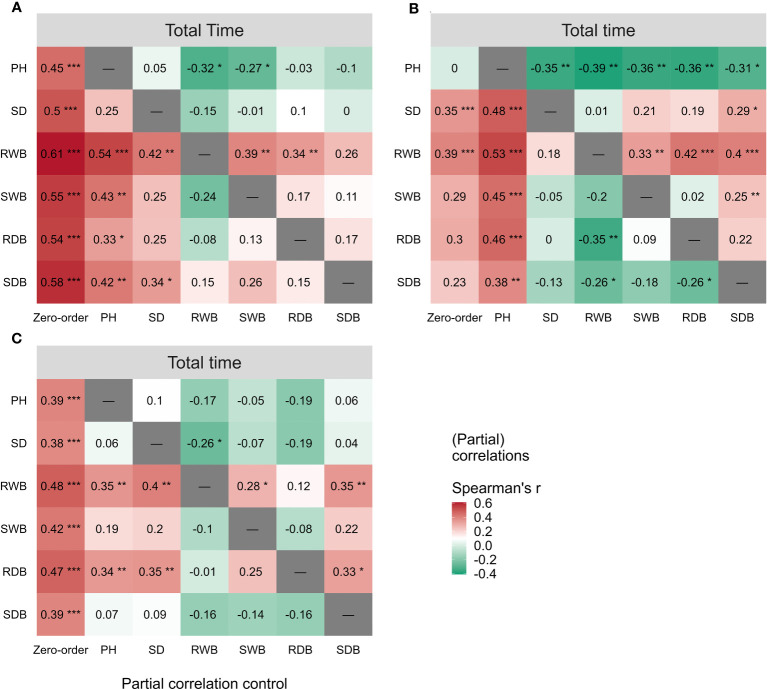
Spearman correlations (First column “Zero-order”) between total time and the six morphological traits (i.e., PH, SD, RWB, SWB, RDB, and SDB) of three species **(A)** CC, **(B)** DC, **(C)** LS. Partial spearman correlations between total time and other five morphological traits by controlling the effect of one morphological trait (last six columns, the “-” or column name indicates the factor that was controlled in the partial correlation analysis). SDB/SWB = shoot dry/wet biomass, RDB/RWB = root dry/wet biomass, SD = stem diameter, PH = plant height. The intensity of colors and numbers indicate the strength of the correlation. Significant levels are: *: P< 0.05; **: P< 0.01; and ***: P< 0.001.

**Figure 6:**
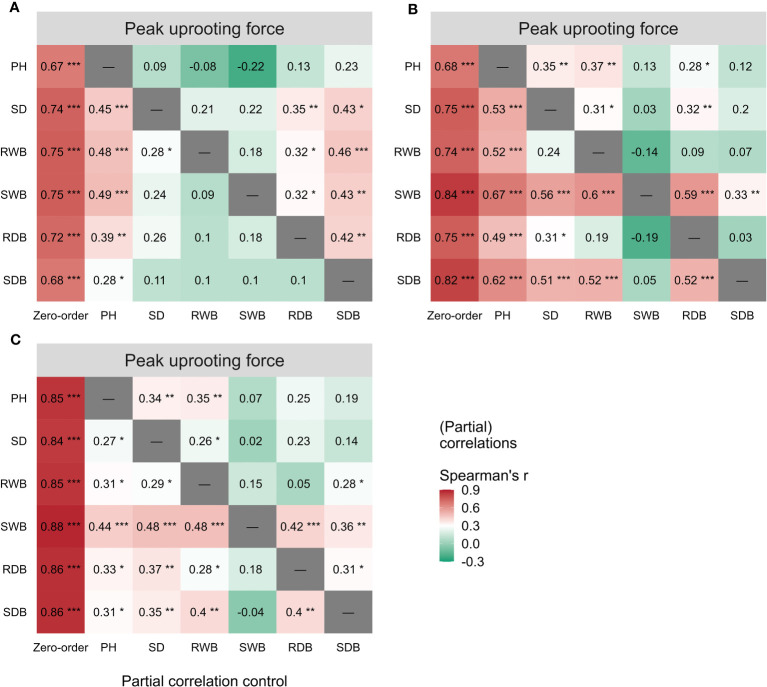
Spearman correlations (First column “Zero-order”) between peak uprooting force and the six morphological traits (i.e., PH, SD, RWB, SWB, RDB, and SDB) of three species **(A)** CC, **(B)** DC, **(C)** LS. Partial spearman correlations between peak uprooting force and other five morphological traits by controlling the effect of one morphological trait (last six columns, the “-” or column name indicates the factor that was controlled in the partial correlation analysis). SDB/SWB = shoot dry/wet biomass, RDB/RWB = root dry/wet biomass, SD = stem diameter, PH = plant height. The intensity of colors and numbers indicate the strength of the correlation. Significant levels are: *: P< 0.05; **: P< 0.01; and ***: P< 0.001.

The outcomes of the Spearman correlation analysis between morphological traits and pullout characteristics are illustrated in **Figure 3**. We found a weak to moderate correlation ( $0.33\leq \;r_{s}\leq 0.61$ ) between the morphological traits and $T_{total}$ for LS and CC. The correlations for CC were higher ( $r_{s}>0.45$ ) than those for LS. There was no significant statistical correlation between most morphological traits and $T_{total}$ in DC (marked with “×” in **Figure 3B**). To explain the difference of correlation between morphological traits and $T_{total}$ in species, we employed partial correlation analysis to eliminate the effect of one morphological trait on other morphological traits. The results are shown in **Figure 5**. The difference between zero-order and partial correlations indicated the dependence degree of the correlation between the given variable (column name) and $T_{total}$ . For DC, negative correlations were found between plant height and $T_{total}$ when we controlled the effects of other morphological traits (**Figure 5B**, first row “PH”), and significant positive correlations were found between other morphological traits and $T_{total}$ when we controlled the effect of plant height (**Figure 5B**, second column “PH”). These results may indicate the reason for the insignificant relationships between most morphological traits and $T_{total}$ in DC (**Figure 5B**, first column “Zero-order”). These results were only seen for DC and not for CC or LS, potentially owing to the special above-ground shape of DC (**Supplementary Figure 2B**). For CC, positive correlations were found between four morphological traits and $T_{total}$ when we controlled the effect of plant height (**Figure 5A**, second column “PH”), while for LS, only two morphological traits were found to be correlated with peak uprooting force (**Figure 5C**, second column “PH”), which implies that the effect of plant height on other morphological traits is higher for LS than for CC. This may be the reason for the higher correlations between morphological traits and $T_{total}$ . The peak uprooting force and morphological traits had a significant positive correlation with $r_{s}\;$ > 0.67 (**Figure 3**). For all three species, the significant positive correlation between the other five morphological traits and peak uprooting force (Spearman’s $r_{s}$ = 0.67 - 0.86, P< 0.001, **Figure 6**, first column “Zero-order” of each plot) was significantly weakened ( $r_{s}$ = -0.22 - 0.22, P > 0.05, **Figure 6**, fourth column “SWB” of each plot) after removing the effect of shoot wet biomass. This suggests that shoot wet biomass was the most important factor affecting peak uprooting force. Notably, when we controlled for the impact of root wet biomass, the correlations between the other five morphological traits and peak uprooting force were insignificant for CC, as depicted in **Figure 6A**. Therefore, in CC, the peak uprooting force was predominantly influenced by root wet biomass and shoot wet biomass. For DC and LS, the peak uprooting force was affected by shoot wet biomass.

### 3.5 Effects of morphological traits on the peak uprooting force

**Figure 7:**
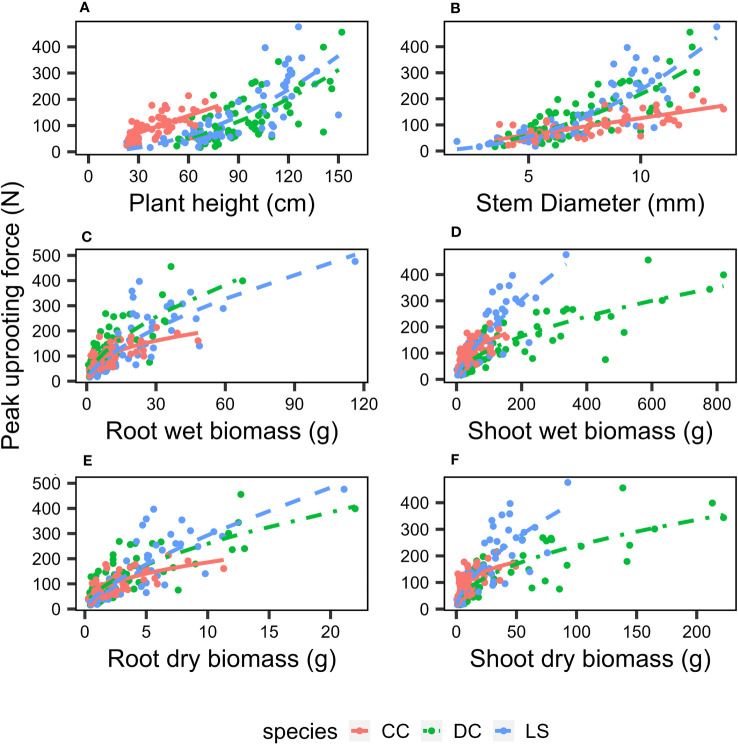
Relationship between peak pullout resistance and morphological traits **(A)** plant height, **(B)** stem diameter, **(C)** root wet biomass, **(D)** shoot wet biomass, **(E)** root dry biomass, **(F)** shoot dry biomass. Different species are marked as: DC (green solid circle), LS (blue solid circle), and CC (orange solid circle), and power regression fit for DC (green solid line), LS (blue solid line), and CC (orange solid line); the coefficients and R^2^ is listed in **Table 1**.

The analysis of the peak uprooting force was conducted as a function of morphological traits due to the substantial positive correlation demonstrated in **Figure 3**. The peak uprooting force increased with six morphological traits (**Figure 7**), following a power function that can be expressed as $F_{R}=m\cdot x^{n}$ , where *m* and *n* are the power regression coefficients (**Table 2**) for morphological traits of different species, and *x* denotes each of the six morphological traits. The coefficient *n* and the power regressions were highly significant with *p*< 0.01 (**Table 2**). However, the coefficient of *m* was not significant between the peak uprooting force with plant height and the stem diameter of DC and CC. The larger the plant (or the traits), the larger the peak uprooting force, with subtle differences between species. The coefficient of determination (R^2^) showed that shoot wet biomass for DC and LS and root wet biomass for LS were the single best variables for predicting the peak uprooting ( $R^{2}=0.72,\;0.75,\;\text{and }0.57$ ). The R^2^ was consistently significantly lower for CC than for DC and LS (**Table 2**). One possible explanation for this is that, for CC, peak uprooting force is mainly affected by shoot wet biomass and root wet biomass, but for DC and LS it is affected by shoot wet biomass (**Figure 6**). The coefficients *m* and *n* ranged between 7.13–25.91 and 0.38–0.71 between the plant diameter and the peak uprooting force of all three species. The R^2^ between root wet biomass and root dry biomass and between shoot wet biomass and shoot dry biomass differed slightly, which may indicate that the moisture content of the shoots and roots slightly affected the fit of the power regression.

**Table 2:** Coefficients, p-values and coefficient of determination for the power regressions between morphological traits and peak uprooting force for three species.

morphological traits	species	a	p (a)	b	p (b)	R^2^	p (y)
plant height (cm)	CC	3.00	0.11	0.93	<0.01	0.43	<0.01
	DC	0.02	0.40	1.94	<0.01	0.50	<0.01
	LS	0.02	0.44	1.94	<0.01	0.60	<0.01
plant diameter (mm)	CC	11.77	<0.01	1.03	<0.01	0.54	<0.01
	DC	3.03	0.02	1.86	<0.01	0.65	<0.01
	LS	1.87	0.08	2.10	<0.01	0.66	<0.01
wet root biomass (g)	CC	45.62	<0.01	0.37	<0.01	0.57	<0.01
	DC	33.97	<0.01	0.59	<0.01	0.61	<0.01
	LS	23.09	<0.01	0.65	<0.01	0.64	<0.01
wet shoot biomass (g)	CC	25.91	<0.01	0.38	<0.01	0.54	<0.01
	DC	8.93	<0.01	0.55	<0.01	0.72	<0.01
	LS	7.13	<0.01	0.71	<0.01	0.75	<0.01
dry root biomass (g)	CC	73.91	<0.01	0.40	<0.01	0.53	<0.01
	DC	69.71	<0.01	0.57	<0.01	0.64	<0.01
	LS	55.47	<0.01	0.72	<0.01	0.68	<0.01
dry shoot biomass (g)	CC	53.39	<0.01	0.31	<0.01	0.46	<0.01
	DC	24.45	<0.01	0.49	<0.01	0.70	<0.01
	LS	24.11	<0.01	0.61	<0.01	0.69	<0.01

“a”,”b” refer to the power regression coefficients; “p(a)”, “p(b)” refer to p-values of significance tests of coefficients “a” and “b”; “p(F_R_)” refer to significance tests of power regressions.

## 4 Discussion

### 4.1 Relationship between aboveground morphological traits and peak uprooting force

**Figure 8:**
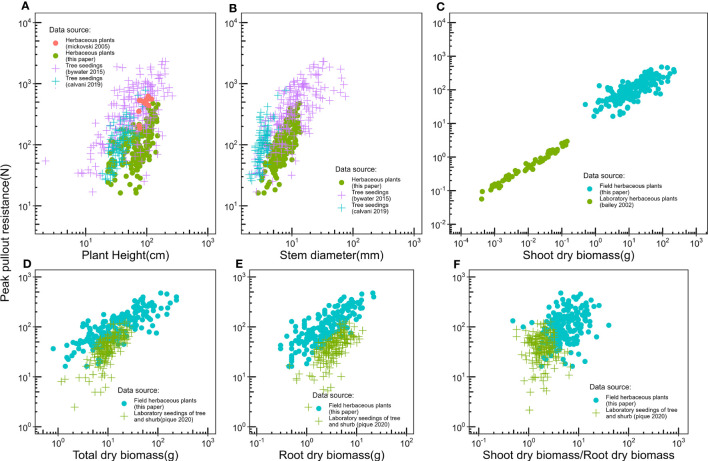
Compare relationships between peak pullout force and **(A)** Plant height, **(B)** Stem diameter, **(C)** Shoot dry biomass, **(D)** Total dry biomass, **(E)** Root dry biomass, **(F)** Shoot dry biomass/Root dry biomass, with the other authors, experimental conditions and the parameters available for each author is listed in **Table 2**. Specially, the plants used in our study, Bailey et al. (2002), and Mickovski et al. (2005) are herb, the plants used in other papers are shrub and tree seedlings.

There is little data on the relationship between plant aboveground morphological traits and peak uprooting force (Bywater-Reyes et al., 2015; Bankhead et al., 2017; Piqué et al., 2020), especially with outdoor experimental data of herbaceous plants. Therefore, in this study, we measured the morphological traits of the above-ground portions of vegetation and their peak uprooting force. We found that peak uprooting force increased with morphological traits of vegetation following a power law function (**Figure 7**), which is consistent with the findings of Bailey et al. (2002) who reported that peak uprooting force increased with shoot dry biomass following a power law function. However, other authors have shown that peak uprooting force increased linearly with plant height (Mickovski et al., 2005), root biomass, and total biomass (Piqué et al., 2020). A possible explanation for the inconsistency in Mickovski et al. (2005) may be that they had a smaller sample size (19 plants) compared to our study. In that study, the range of plant height of vetiver grass was 0.74–1.08 m with a SD 0.04 m. The SD is smaller compared to our study (SD of 0.14, 0.24, and 0.29 m for CC, DC, and LS, respectively), which indicates only a small intraspecific difference of plant height. The inconsistency with Piqué et al. (2020) may be that the species in their study were seedlings of shrubs and trees, aged about 1 year, which is much younger than the mature individuals. We compared the relationship between peak uprooting force and morphological traits with other authors (Bailey et al., 2002; Mickovski et al., 2005; Bywater-Reyes et al., 2015; Calvani et al., 2019; Piqué et al., 2020); their experimental conditions and available parameters are listed in **Table 3**, including experimental location, plant growing environment, plant species, plant type, plant age, uprooting direction, number of samples, and available parameters. The results were shown in **Figure 8**, all the panels are presented in log-log coordinates. The species in Bailey et al. (2002) and Mickovski et al. (2005) are herbaceous species, while those of other studies are seedlings of shrub and tree. The plant growing environment in Bailey et al. (2002) and Piqué et al. (2020) were indoor laboratories, while that of the other studies was in the field. Herbaceous plants had a lower peak uprooting force than tree seedlings with the same height and stem diameter (**Figures 8A, B**). Several factors could explain this observation. Firstly, seedlings of shrub and tree have a longer root length than herbaceous plants of the same plant height and stem diameter. Secondly, the lateral pullout tests in Mickovski et al. (2005); Bywater-Reyes et al. (2015), and Calvani et al. (2019) showed a greater peak uprooting force than was found in the vertical uprooting tests of this study (Yang et al., 2021). Finally, soil type is also known to affect peak uprooting force by affecting the root soil interfacial friction (Schwarz et al., 2011). For the same root dry biomass or total dry biomass, trees had a lower peak uprooting force than herbaceous plant (**Figures 8D, E**), which may be caused by the limited space for tree seedling growth in Piqué et al. (2020) (**Figures 8D, E**). A strong linear relationship was recorded between the shoot dry biomass and peak uprooting force. Although the plant species and the experimental conditions in our study differed from those in (Bailey et al., 2002), our findings suggest that the linear relationship between the logarithmic peak uprooting force and the logarithmic shoot dry biomass was less affected by the experimental conditions. It is obvious that the peak uprooting force increased with the increasing morphological traits of shoot part (**Figures 8A–C**), which indicated that it is possible to estimate peak uprooting force by aboveground morphological traits. However, abovementioned studies on peak uprooting force have primarily focused on specific growth stages of plants, overlooking the effects of overall plant growth. In general, for annual herbaceous plants, morphological traits experience an initial phase of increase due to plant growth, followed by a subsequent phase of decrease as the plants enter senescence. These changes in morphological traits can consequently lead to variations in peak uprooting force at different growth stages. For instance, in the case of *Chenopodium album* and *Setaria viridis*, the average root area ratio and average root additional cohesion showed an increasing trend as the growth period extended from 14 days to 131 days after germination, indicating a positive correlation. However, the tensile force of individual roots in these herbaceous plants was observed to be the lowest on day 14 and day 21, respectively, and showed a significant increase after an additional 7 days of growth, with subsequent growth having less pronounced effects (Hao et al., 2023). Conversely, Mao et al. (2023) investigated the influence of alfalfa roots on the additional cohesion of loess and found that it exhibited an initial increase followed by a decrease as alfalfa underwent growth from 60 days to 150 days after germination. This discrepancy in root additional cohesion between the two studies may be attributed to different plant species or variations in the growth period. To our knowledge, there is limited research exploring the variation in peak uprooting force of annual herbaceous plants from germination to senescence. Therefore, further investigations are encouraged to examine the changes in peak uprooting force throughout the entire life cycle of annual herbaceous plants.

**Table 3:** Summary table containing the experimental conditions and the parameters available for every data set used in **Figure 8**.

	Bailey et al. (2002)	Mickovski et al. (2005)	Bywater-Reyes et al. (2015)	Calvani et al. (2019)	Piqué et al. (2020)
Plant species	Arabidopsis	Vetiveria zizanioides	Populus, Salix, Tamarix	Salix	Aristotelia chilensis, Cryptocarya alba, Escallonia illinita, Fuchsia magellanica,Lithraea caustica, Maytenus boaria, and Quillaja saponaria,Acacia dealbata and Acacia melanoxylon
Plant type	Herb	Herb	Tree seedings	Tree seedings	Shurb and tree seedings
Plant growth conditions	Laboratory	Outdoor(not monitored)	Outdoor(not monitored)	Outdoor(not monitored)	Laboratory
Cultivation time/plant age	5-9 weeks	NA	1-5 years old	1-3 years old	1 years old
Type of sediment	Sand and gravel	Marl	Sand and gravel	Gravel	Sand, gravel,cobbles
Type of uprooting	Vertical pull test	Lateral pull test	Lateral pull test	Lateral pull test	Vertical pull test
Uprooting location	Single batch	Almudaina	Bitterroot River, Bill Williams River, Santa Maria River	Ombrone Pistoiese River	Clastic growing bags
Number of samples	60	16	198	93	174
Parameters available	Shoot dry biomass, Peak pullout force	Height, Peak pullout force	Height, Basal diameter, Peak pullout force	Height, Stem diameter	Root dry biomass, Total dry biomass, Aerial/Root dry biomass

### 4.2 Factors affect the estimation of critical flow velocity

Equation (6) enables us to anticipate the critical flow velocity ( $U_{c})$ at which a submerged plant will be dislodged as a result of Type I uprooting. To derive equation (6), we combined two allometric equations, which causes a decrease in R^2^. In this study, we just gauged plant height, stem diameter, and shoot wet/dry biomass, not including frontal area of aboveground part. In actual application, we recommend using the frontal area of the aboveground part of the vegetation instead of the vegetation height, which to avoids the use of two allometric equations. With this approach, Equation (6) can be transformed to $U_{c}=\;\sqrt{2dc\cdot A_{f}^{e-1},\;}$ where d, e is the coefficients in the power law function $F_{R}=\;d\cdot A_{f}^{e}$ , c is a coefficient in Equation (6). This power law function is verified according to the data downloaded from Bywater-Reyes et al. (2015) (**Supplementary Figure 4**). According to the power law functions between frontal area and peak uprooting force of *Populus*, *Tamarix*, and S*alix* (**Supplementary Figure 4**). We found the coefficient *e* is 0.69, 0.42, and 0.96 for *Populus*, *Tamarix*, and S*alix*. The result shows $U_{c}\;$ decreases with the increase of $A_{f}$ , which means as the frontal area of a plant increases, the velocity needed to uproot the submerged plant decreases.

To derive equation (6), we equated $F_{R}=\;F_{D}$ . However, it is crucial to acknowledge that several factors can affect $F_{R}$ or $F_{D}$ , which may result in an overestimation or underestimation of the critical velocity. These factors include the flexibility of the plant, the presence of scour and deposition around a plant or plant patch, and the direction of pullout. These variables introduce variability and can potentially influence the precision of estimating the critical velocity.

As outlined in the introduction, previous studies by Edmaier et al. (2011) have categorized riparian vegetation uprooting during floods into type I and type II. Additionally, Bywater-Reyes et al. (2015) further distinguished type II uprooting mechanisms as type IIa and type IIb, based on the different sources of erosion around the plant stem. Type IIa uprooting involves self-induced scouring resulting from fluid-obstacle interaction. This leads to the formation of a horseshoe vortex upstream and around the stem, promoting erosion (Perona et al., 2012). Studies have indicated that this scouring effect reduces the pullout force (Bywater-Reyes et al., 2015; Edmaier et al., 2015). To address this, researchers have proposed substituting the actual plant root length with the critical root length in calculating the peak pullout force (Bau et al., 2019). Consequently, they have put forward a conceptual model and a new physical equation to predict the flow and bed erosion conditions that contribute to plant uprooting (Calvani et al., 2019). In our study, we focused solely on type I uprooting and did not consider scour around the plant stem. Consequently, this led to an overestimation of the critical velocity. Type IIb uprooting, on the other hand, is predominantly driven by larger-scale scour processes that scale with the bar length or river width. At this scale, the influence of individual plants is limited and is taken into account within a vegetation patch. Vegetation patches have complex effects on flow patterns, scour, and deposition. Within and behind the vegetation patch, particle deposition is often enhanced due to reduced mean and turbulent velocities (Chen et al., 2012; Nepf, 2012). Conversely, in the front and lateral areas of the patch, scouring and resuspension can intensify due to induced river width and increased flow velocity (Yagci et al., 2016; Huai et al., 2021). However, the current research lacks an understanding of how erosion and deposition specifically impact the pullout force of an entire vegetation patch. It remains challenging and significant to determine the influence of scour or deposition on the critical flow velocity of the entire vegetation patch. Additionally, the flexibility of plants plays a vital role in their interaction with flowing water (Verschoren et al., 2016). The pliability of leaves and stems allows plants to modify their shape, frontal area, and size in response to flow forces. This flexibility enables them to optimize the trade-off between drag and dynamic reconfiguration (Vogel, 1984; Siniscalchi and Nikora, 2013). As the flexibility of plants increases, the effective frontal area decreases, resulting in a reduction of the drag force. This reduction in $F_{D}$ can be accounted for by incorporating the Vogel exponent (λ) into equation (1), where the velocity term $U^{2}$ is replaced with $U^{2+\lambda }$ (Vogel, 1984). The Vogel exponent represents the plant’s ability to bend and pronate, with values ranging from 0 (rigid blade) to -2 (extremely flexible), depending on the vegetation type (Nepf, 2012). In this study, we did not consider the flexibility of plants when estimating the critical flow velocity, which may lead to an underestimation.

Furthermore, the direction of the pullout test also impacts $F_{R}$ . Previous studies have adopted various pullout test directions, including vertical (Bailey et al., 2002; Edmaier et al., 2014; Piqué et al., 2020; Yang et al., 2021), lateral (Bywater-Reyes et al., 2015; Yang et al., 2021), and horizontal (Mickovski et al., 2005). Among them, only (Yang et al., 2021) investigated the influence of two pullout test directions on the pullout force of alfalfa concurrently. In their experiment, they conducted pullout tests at 90° and 45° angles, and their findings revealed that the pullout direction significantly affected the pulling force of alfalfa roots. They suggested that the vertical pullout test provides a safety margin and is recommended for determining the peak pullout force. Therefore, employing a vertical pullout test may result in an underestimation of the critical flow velocity.

In summary, the accurate estimation of critical flow velocity and pullout force in plants presents a complex challenge. Further research is necessary to gain a deeper understanding of these factors and establish quantitative measures that can enhance our prediction of plant responses to flowing water. This continued investigation will contribute to advancing our knowledge of plant hydraulics in both natural and engineered environments.

### 4.3 Complexity of the *in-situ* environment

Our experiment was conducted on a bar located in the Minjiang River. Although an *in-situ* pullout test does provide a realistic pulling and failure mechanism for the soil-root system (Vergani et al., 2016) considering root tortuosity and root branching (Dazio et al., 2018), the *in-situ* test environment can be complex and uncontrollable, involving factors such as soil water content, soil type, soil heterogeneity, plant growth stage, and plant intraspecific differences. These variables affect the morphological traits and pullout characteristics of plants. Soil water content causes a noticeable effect in soil-root interactions, with roots in dry soils requiring a greater uprooting force than those in wet soils (Schwarz et al., 2011), which means peak uprooting force decreases with increasing soil water content (Fan et al., 2021). The influence of soil moisture on peak uprooting force depends on soil type because soil moisture can affect the effective normal stress and apparent cohesion (Schwarz et al., 2010). In our study, the altitude of the bar ranged from 418.2–419.3 m. From the lowest to the highest point, we tested three soil samples which had water contents of 55.1%, 53.6%, and 48.9% respectively. This is a slight difference compared to the range of water content (12.1–30.6%) in Fan et al. (2021), which has few effects on peak uprooting force in our study. During flood, plants were emerged or submerged, the soil can be considered fully saturated. Therefore, the peak uprooting force measured in the unsaturated condition in this experiment may result in an overestimation.

The soil on the bar was mainly composed of gravels and cobbles, comprising 92% of the mass fraction of the soil materials. The soil had a high level of heterogeneity because of the presence of large gravels on the surface and within the soil. Soil type and soil heterogeneity may affect root system development (Ennos, 2000). The cross section of the root grown on a uniform substrate such as sand was closer to a regular round form. When grown on a non-uniform substrate, such as gravel, root systems have an irregular cross-section (Oplatka and Sutherland, 1995). The irregular cross-section of the roots is prone to stress concentration when the root system is stressed. These factors may be responsible for the large intraspecific difference of morphological traits, peak uprooting force. We sampled enough samples to reduce to effects of soil water content, soil heterogeneity, and groundwater table.

In July 2020, we observed a vegetation removal event on this bar during flooding, in which the flood removed all plants from the bar (**Figure 1B**). Our experiments were conducted in May 2021, during which all the plants had undergone only one spring of growth. Since the temperature across different positions within the river bar remains consistent, we believe that there is minimal variation in the germination time among individuals of the same species. Additionally, distinguishing the age of different individuals within the same plant species is challenging for us. Consequently, in this experiment, we did not differentiate the age of the vegetation samples.

The abovementioned factors will result in intraspecific variation in the morphological traits and pullout characteristics of plants. The data of morphological traits and pullout characteristics of vegetation showed extreme values, and the distribution was non-normal. Therefore, the results of this study were constrained by species, duration, and the conditions of the *in-situ* environment. The results remain to be verified for other species. Further research is needed to investigate the effects of soil type and soil heterogeneity, and growth period on morphological traits and the pullout characteristics of vegetation in a controlled environment.

## 5 Conclusions

To investigate the uprooting force of riparian vegetation in a typical riverine environment, we conducted a series vertical uprooting tests of three dominant species on a bar, and measured the plant morphological traits and peak uprooting force. The results showed that morphological traits were different among species; however, all three species had a similar median peak uprooting force. We analyzed the correlation between the morphological traits and peak uprooting force, and found (i) a strong correlation between arbitrary two morphological traits of CC and LS; (ii) a moderate to strong correlation between arbitrary two morphological traits of DC, and (iii) a moderate to strong correlation between any of the morphological traits and peak uprooting force. Finally, we fitted power law functions between morphological traits and peak uprooting force. The power law functions, are analogous to allometric equations, were found to provide an easy and fast means of estimating peak uprooting force according to plant aboveground morphological traits. Despite some limitations (recall Section 4.3), this study is representative of the common uprooting process of riparian vegetation in a typical riverine environment, and provides a simple way to estimate the peak uprooting force of a plant. The present experiment has potential to be reproduced for other soil types and other annual vegetation, which is more common in the riverine zone and more susceptible to flood disturbances compared to shrubs and trees. It is also hoped that future studies will provide as much information as possible about the environment of the experimental site, including but not limited to site location, soil conditions, climate, and rainfall, to facilitate comparison by other authors.

## Data availability statement

The datasets presented in this study can be found in online repositories. The names of the repository/repositories and accessionnumber(s) can be found below: https://doi.org/10.5281/zenodo.6476708.

## Author contributions

SH planned and designed the research. YZ wrote the manuscript and performed experiments. WL analyzed data. All authors contributed to the article and approved the submitted version.
