# Supplementary material for: Allometric equations for estimating peak uprooting force of riparian vegetation
Source: Front Plant Sci. 2023 Jul 3;14:1192486. doi: 10.3389/fpls.2023.1192486 (PMC10352110; doi:10.3389/fpls.2023.1192486)
Supplement: Supplementary file 1 [file DataSheet_1.docx]

Supplementary Material

Allometric Equations for Estimating Peak Uprooting Force of Riparian Vegetation

**Yi Zhang, Wei Liu, Siming He***

*** Correspondence:** Siming He: hsm@imde.ac.cn

#
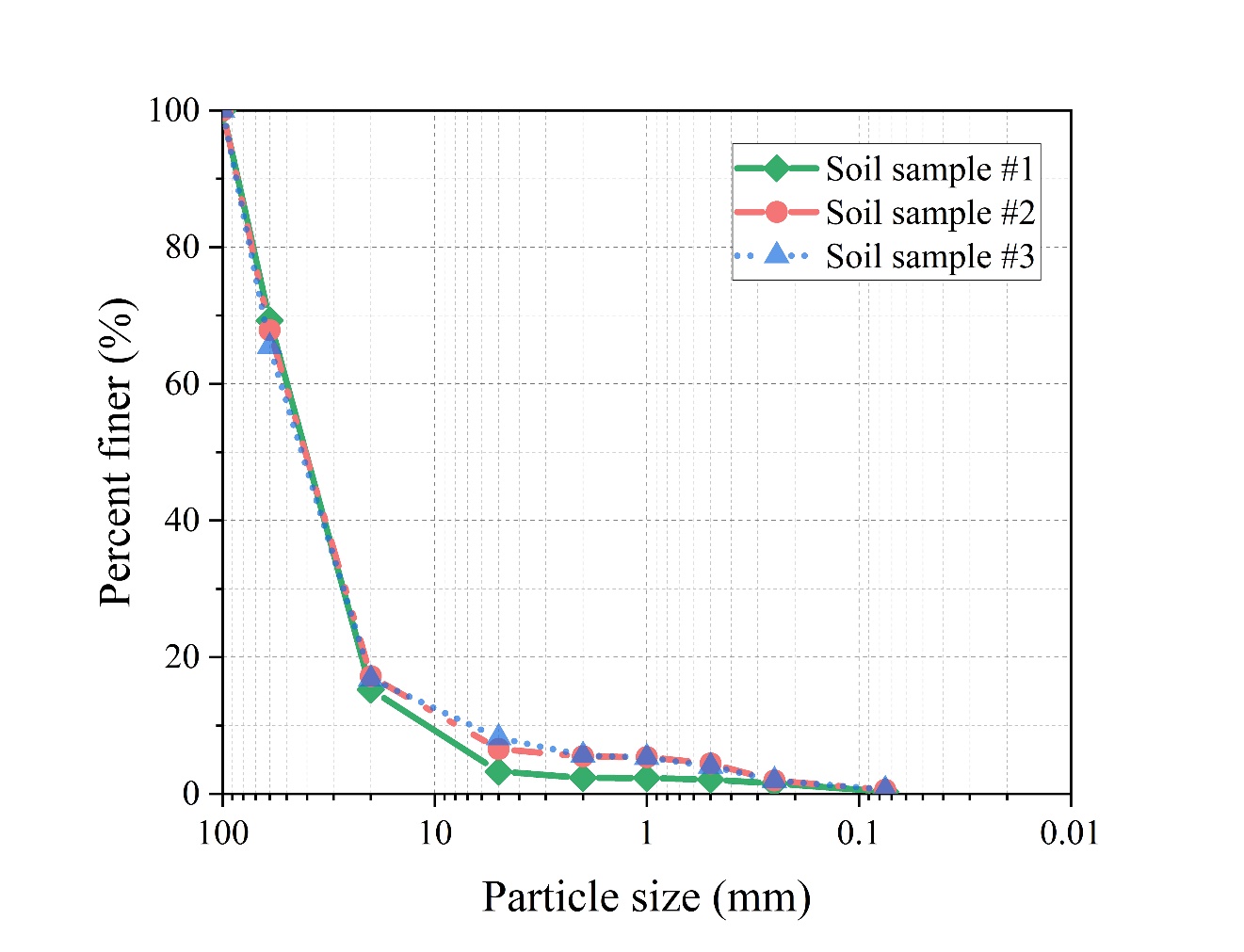
Supplementary Figures

## Supplementary Figure S1. The soil particle distribution curve of three samples at the bar.


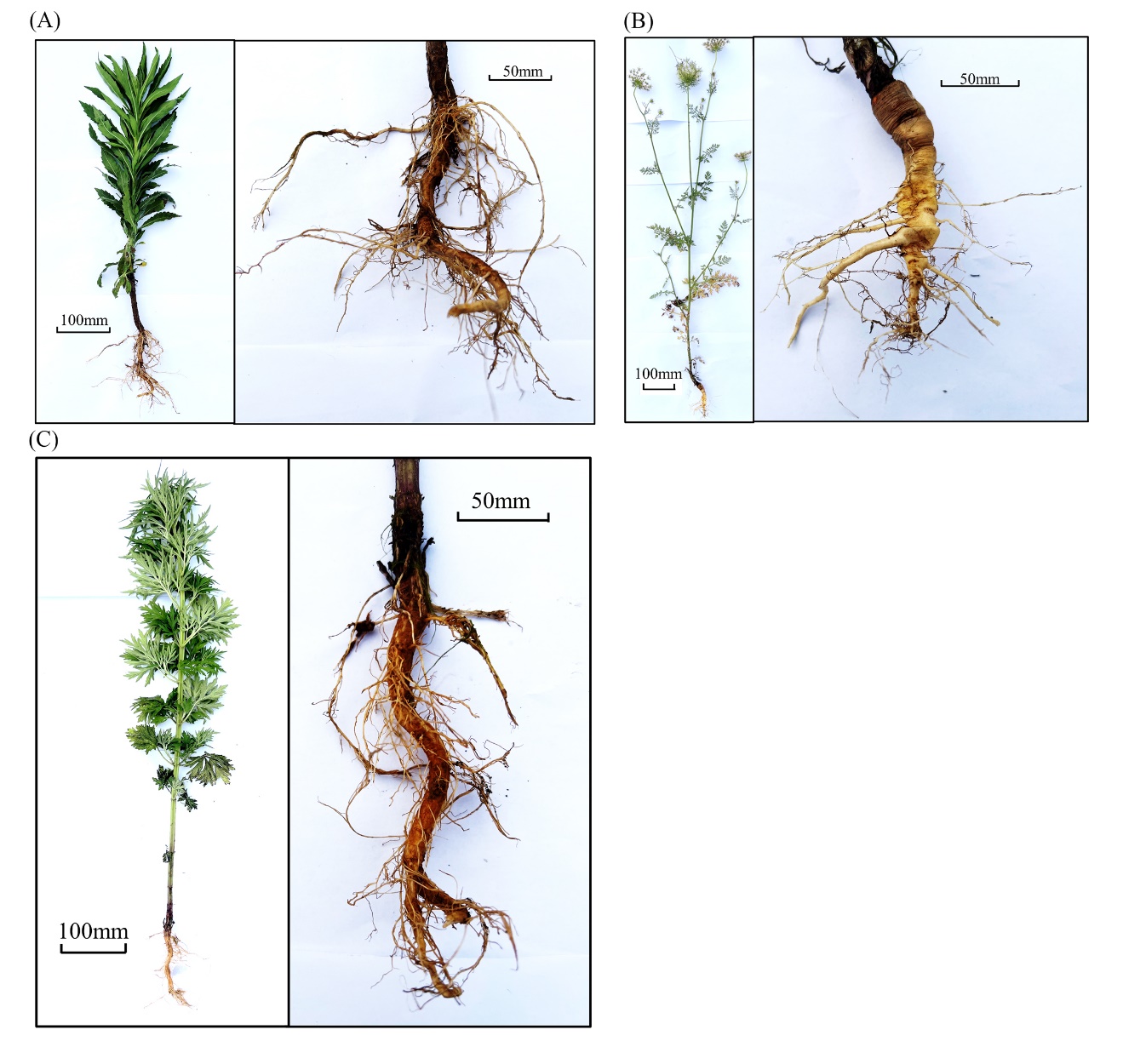
**Supplementary Figure S2.** Whole part and root system of three dominant species: (A) CC; (B) DC; (C) LS.

**Supplementary Figure S**
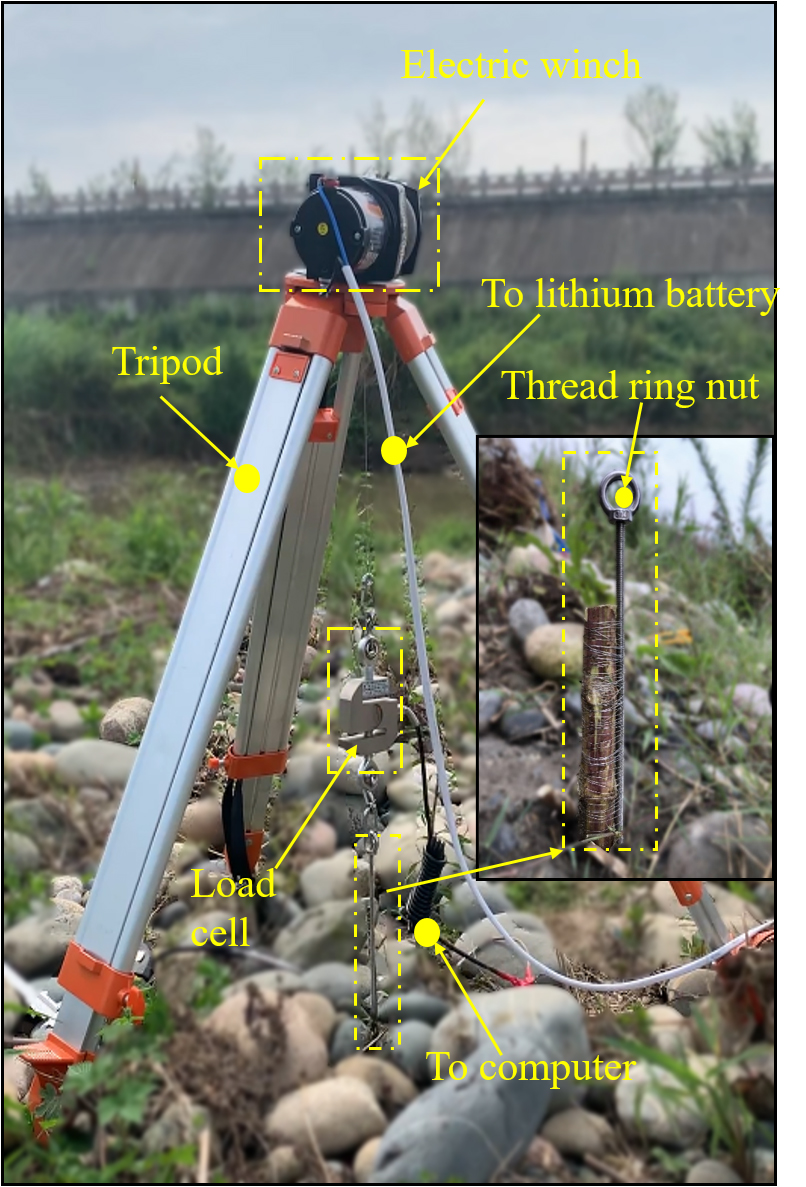
**3.** An uprooting mechanism used in our experiments, it is mainly consisted of tripod, load cell, and electric winch.


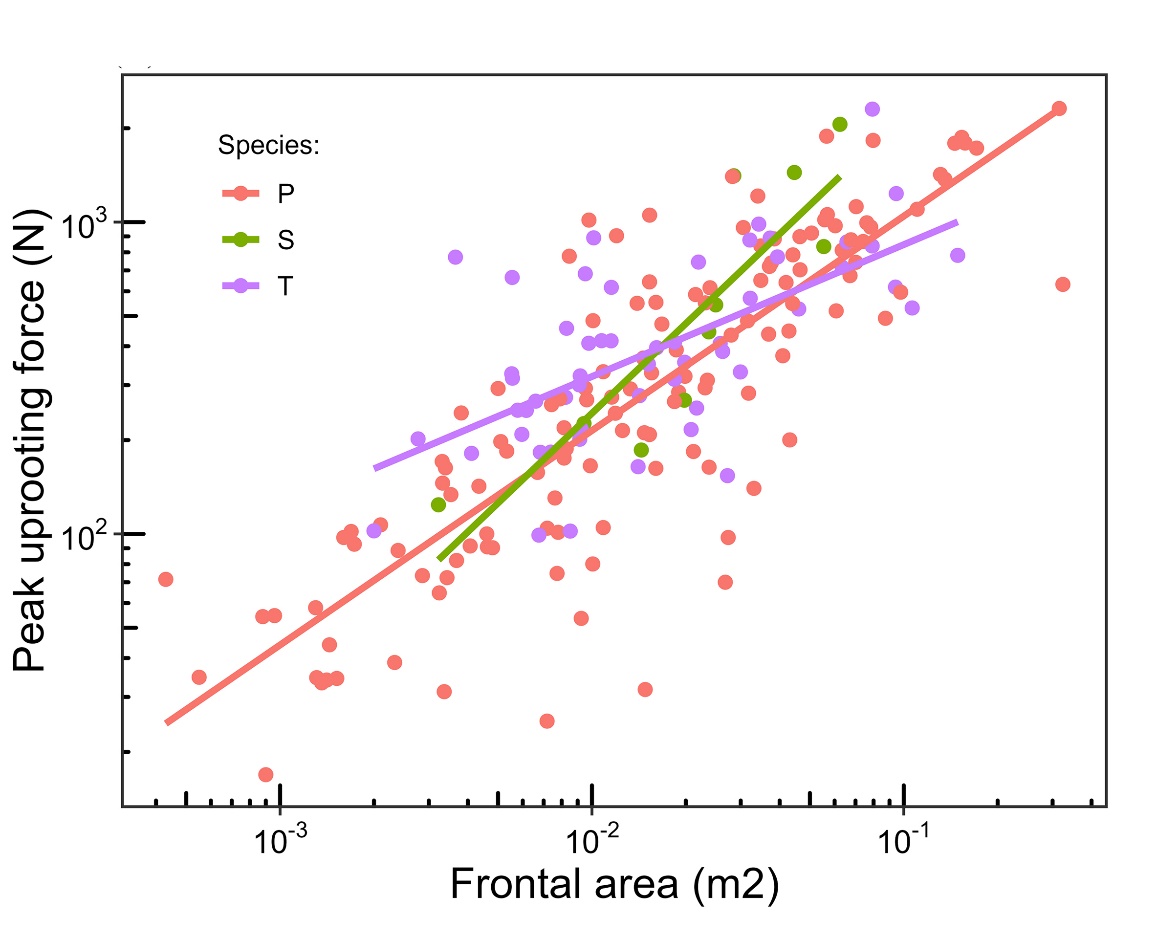
**Supplementary Figure S4.** Relationships between peak uprooting force and frontal area. Data is provided by Bywater‐Reyes et al. (2015). All the panels are presented in log-log coordinates. P means *Populus*, T means *Tamarix*, and S means S*alix.*

**References**
